# Femoral Neck Fractures: Incidence, Reasons, and Risk Factors of Conversion From Hemiarthroplasty to Total Hip Arthroplasty

**DOI:** 10.5435/JAAOSGlobal-D-24-00312

**Published:** 2025-05-13

**Authors:** Michael J. Gouzoulis, Rajiv S. Vasudevan, Stephanie V. Kaszuba, Anthony E. Seddio, Lee. E. Rubin, Jonathan N. Grauer, Mengnai Li

**Affiliations:** From the Department of Orthopaedics and Rehabilitations, Yale School of Medicine, New Haven, CT.

## Abstract

**Background:**

There is controversy over the choice of hemiarthroplasty (HA) versus total hip arthroplasty for treatment of femoral neck fractures in geriatric patients, especially those who are relatively healthy and active. A concern for selection of HA is that the patients may later require conversion to THA.

**Methods:**

All geriatric patients with femoral neck fractures who underwent HA were identified in the national PearlDiver data set. Patients were required to have 5 years of follow-up, and incidence of conversion was determined. Univariable and multivariable analyses were conducted to determine factors associated with conversion. The timing of conversion and reasons for conversions were determined.

**Results:**

A total of 7,501 patients were identified with femoral neck fractures who underwent initial treatment with HA. Of those, 173 (2.3%) underwent conversion to THA within 5 years. On multivariable analysis, conversion was associated with patients being younger than 75 years (odds ratio: 1.64, *P* = 0.002) and having a higher Elixhauser Comorbidity Index (odds ratio: 1.04 per point, *P* = 0.018). Of the conversions performed over a five year period, 109 patients (63.0%) were within the 1st year. The most common reason for conversion to THA was degenerative causes (67.6%).

**Conclusions:**

Overall, there was a low rate (2.3%) of conversion from HA to THA for those who were tracked for 5 years after HA for femoral neck fracture. This low conversion rate is supportive that HA offers a durable answer for most patients for whom this treatment choice is pursued.

**Levels of Evidence:**

III.

Hip fractures are common among geriatric patients, with 300,000 occurring each year in the United States.^1^ With the aging population, it is expected that the incidence of hip fractures will continue to increase.^2^ Femoral neck fractures are one of the more common types of hip fracture patterns, making up over 50% of all hip fractures.^3,4^ For displaced fractures, hemiarthroplasty (HA) or total hip arthroplasty (THA) is frequently considered and discussed regarding which is the best treatment option.^5^

Hemiarthroplasty is the second most common treatment for femoral neck fractures.^6^ This has the advantage of addressing the fracture while limiting the scope of surgical intervention/complications^7-9^ and minimizing the risk of dislocation.^10-12^ However, HA has the risk of subsequent degenerative pain due to acetabular chondrolysis and disease progression, especially for more active patients with preexisting acetabular wear. In these situations, THA may be considered.

While some literature has suggested that patients treated with THA may have improved functional outcomes,^13,14^ recent randomized control trials demonstrated no clinically significant improvement in function or quality of life within 2 years after surgery.^12,15^ In addition, HA and THA have similar 1-year revision rates.^15-17^ Nonetheless, a concern related to the choice of HA is the possible need for conversion to THA over time.^13,18^ For this reason, some surgeons argue for initial management of femoral neck fractures in those who are relatively healthy and active with THA to avoid having to potentially convert a HA to a THA.^19^

This study makes use of a large national data set to look at the incidence, reasons, and risk factors of HA conversion to THA. It was hypothesized that, while there is a risk of conversion from HA to THA after fracture, the overall risk would be low.

## Methods

### Study Cohort

This study made use of the 2015-Q3 2022 M165Ortho PearlDiver Mariner Patient Claims Database. This is a national insurance claims data set that uses patient information from the inpatient and outpatient setting. This data set has been frequently used in the trauma and joint literature.^20-23^ Our institutional review board has determined that studies using PearlDiver are exempt from requiring review because all data are outputted in an aggregated format and are deidentified.

All geriatric (65 years and older) patients with a femoral neck fracture were identified using International Classification of Diseases diagnostic codes. Patients who were treated with HA were identified with Current Procedural Terminology (CPT) code CPT-27236. Because this code is unspecific between HA and pinning, ICD-10 diagnostic codes for the presence of an artificial hip joint were also used concurrently to identify patients who specifically received a HA for management. To understand the incidence of HA versus THA for initial management of femoral neck fractures, rates of primary management of HA or THA were also determined.

### Conversion to Total Hip Arthroplasty

Patients were followed for 5 years after their surgery for conversion of HA to THA. They were required to have at least 5 years' worth of follow-up; as a result, this likely selected for a cohort with longer survival and an overall healthier patient population. Total hip arthroplasty conversion was determined using CPT codes CPT-27130 and CPT-27132. In addition, laterality of the conversion was determined using ICD-10 codes to ensure that the THA conversion occurred on the same side as the HA.

The timing of conversion after the primary index procedure was determined. In addition, the reason for revision surgery was determined. This was categorized into the following groups: degenerative, dislocation, periprosthetic infection, periprosthetic fracture, and other (including mechanical complications).

### Data Analysis

Patient demographics of those undergoing HA were tabulated and categorized into two groups depending on whether they did or did not undergo later conversion to THA. The following variables were included: age, sex, Elixhauser Comorbidity Index (ECI), and obesity. ECI is a common comorbidity index that demonstrates the overall comorbidity of a patient and has been validated in the orthopaedic trauma literature.^24^ Univariable analysis was used to determine whether there was a significant difference in each variable between those who did and did not undergo conversion. Chi-square tests were used to compare sex and obesity while the standard *t* test was used to compare age and ECI.

Multivariable logistic regression was used to determine independent predictors of undergoing conversion. Odds ratios (ORs) and 95% confidence intervals (CIs) were determined. The multivariable logistic regression controlled for age, sex, ECI, and obesity.

All statistical analyses were conducted using RSuite statistical software that is built into PearlDiver data set (PearlDiver Technologies). All figures for this study were made using GraphPad Prism 10 (GraphPad Software). Significance was determined using a cutoff of *P* < 0.05.

## Results

### Patient Characteristics

A total of 10,655 patients with femur neck fracture managed with either HA or THA were identified. From that cohort, the initial management was THA for 3,155 (29.6%) and HA for 7,501 (70.4%). Of those undergoing HA, 173 patients (2.3%) underwent a revision surgery to convert their HA to a THA over the course of the study period.

On univariable analysis, HA patients who underwent conversion to THA were more likely to be younger than 75 years (41.6% vs. 30.0%, *P* = 0.001), have higher ECI (8.0 vs. 6.2, *P* < 0.001), and be obese (28.9% vs. 21.7%, *P* = 0.029; Table1). On multivariable analysis, there were increased odds of undergoing conversion to THA for those patients younger than 75 years (OR: 1.64, 95% CI, 1.20 to 2.23, *P* = 0.002) or with higher ECI (OR: 1.04 per point, 95% CI, 1.01 to 1.08, *P* = 0.014; Table 1).

**Table 1:** Patient Characteristics and Predictive Factors of Conversion to Total Hip Arthroplasty After Hemiarthroplasty

Factor or Variable	No Conversion	Conversion to THA	*P*	Odds Ratio (95% CI)	*P*
	7,328 (97.7%)	173 (2.3%)			
Age (SD)	75.4 (4.0)	74.8 (4.4)	0.084	1.64 (1.20-2.23)	**0.002**
Younger than 75	2,200 (30.0%)	72 (41.6%)	**0.001**		
Sex			0.307		
Female	5,623 (76.7%)	139 (80.3%)		Ref	**Ref**
Male	1,705 (23.3%)	34 (19.7%)		0.72 (0.49-1.06)	0.109
ECI	8.0 (4.3)	6.2 (3.7)	**<0.001**	1.04 per point (1.01-1.08)	**0.014**
Obesity	1,588 (21.7%)	50 (28.9%)	**0.029**	1.21 (0.84-1.71)	0.297

ECI = Elixhauser Comorbidity Index; THA = total hip arthroplasty, bolding incdicates significance of *P* < 0.05.

### Timing and Reasons for Revision Surgeries

**Figure 1:**
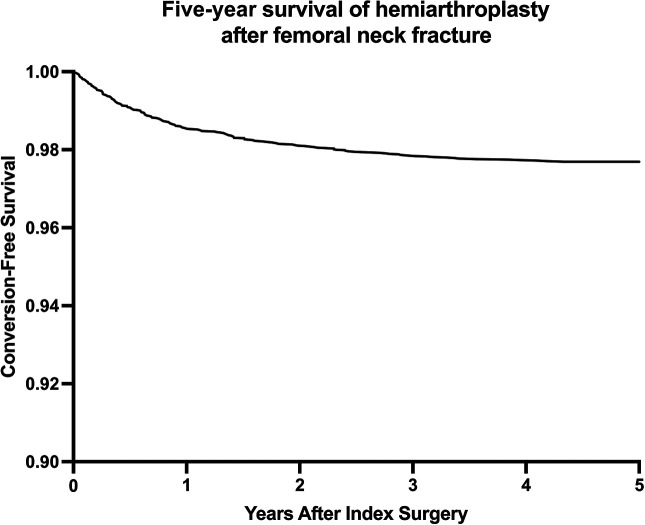
Graph demonstrating the 5-year survival of the hemiarthroplasty (HA) implant is shown. After 5 years, 2.3% of patients were converted to a total hip arthroplasty (THA).

Over a 5-year period, 173 patients (2.3%) underwent conversion to THA. Of all revision surgeries, over half (109 patients, 63.0%) occurred within the first year after surgery while from years 4 to 5, 12 patients (6.9% of all conversions) underwent conversion to THA (Figure 1).

**Figure 2:**
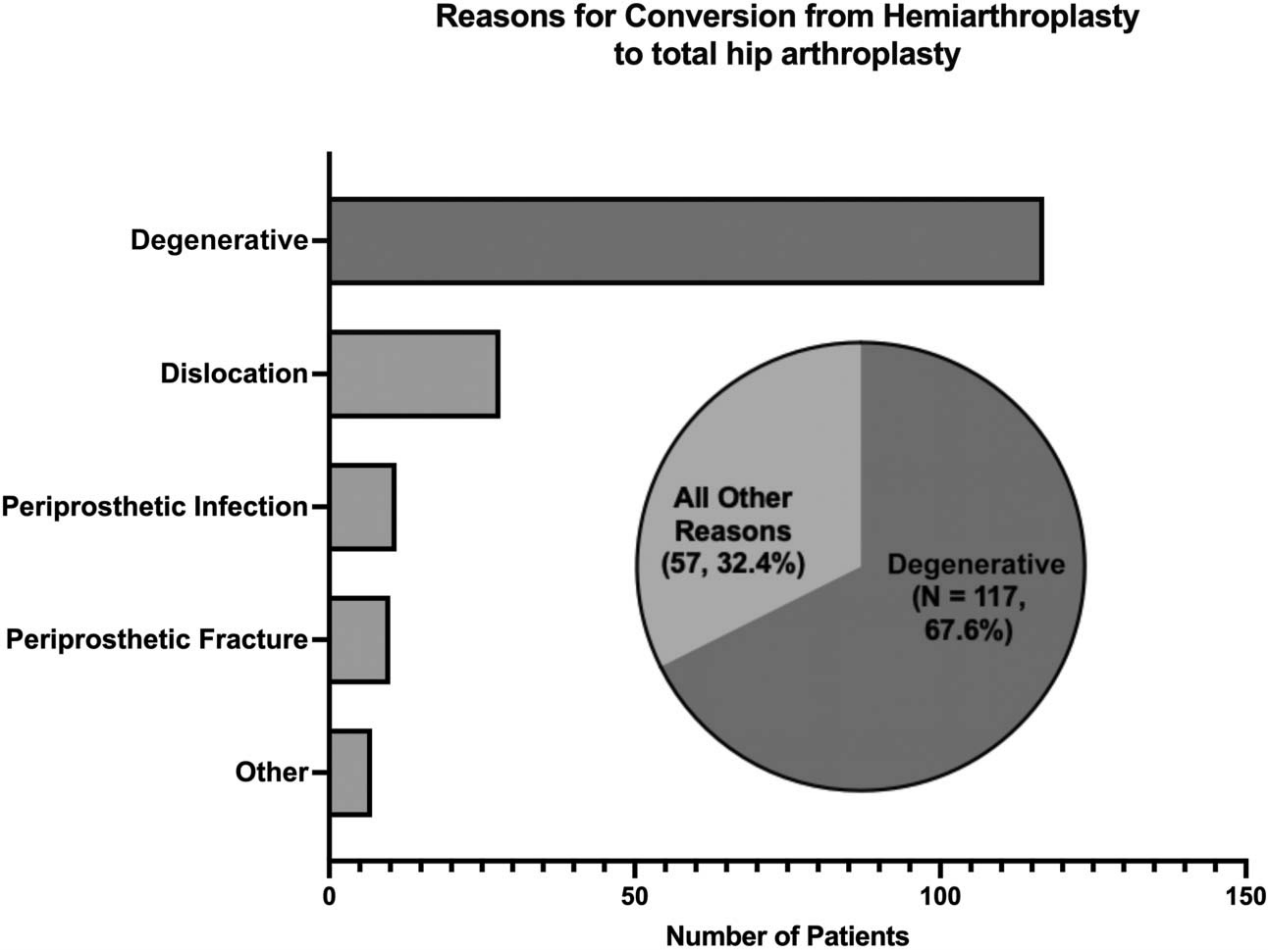
Chart demonstrating reasons for requiring conversion from hemiarthroplasty (HA) to total hip arthroplasty (THA) after femur neck fracture.

Most conversions to THA occurred because of degenerative causes of the hip joint (117 patients, 67.6%). Besides degenerative reasons, we found the following in descending order from most to least common: dislocation (28 patients, 16.2%), periprosthetic infection (11 patients, 6.4%), periprosthetic fracture (10 patients, 5.8%), and other mechanical complications (7 patients, 4.0%; Figure 2).

## Discussion

This study is one of the largest assessments of rates, reasons, and risk factors of HA conversion to THA using a large United States–based database. In this cohort, we found a low rate of 5-year conversion from HA to THA (2.3%), with most conversions indicated for a degenerative etiology (67.6%). Furthermore, patient characteristics associated with conversion were identified.

Indications to perform HA versus THA in the setting of femoral neck fractures are controversial and have been the subject of much debate and investigation over the recent decades. Hemiarthroplasty has been associated with reduced odds of dislocation, decreased surgical time, and decreased blood loss in several recent studies and meta-analyses.^17,25^ However, several randomized controlled trials (RCTs) have suggested that THA is associated with greater quality-of-life and satisfaction metrics, lower revision surgery rates, and lower costs.^26-28^ In 2021, the AAOS downgraded their clinical practice guideline recommendations from strong to moderate for unstable femoral neck fracture for selection of THA, noting small sample size, risk of increase in complications, and potential selection bias.^29^ Interestingly, the recent HEALTH trial in 2019 found no significant difference in postoperative revision rates, with a significant but clinically irrelevant difference in postoperative quality-of-life metrics in favor of THA over a 2-year period.^12^

A subsequent meta-analysis of RCTs in 2020 by Ekhtiari et al^30^ investigating HA versus THA in patients older than 50 years found no difference in revision rates, periprosthetic fracture, mortality, postoperative function, and dislocation rates up to 5 years postoperatively. However, a retrospective review of the Dutch arthroplasty register including all HA and THA procedures for hip fractures found that HA had a lower 1-year revision rate compared with THA.^31^

While some studies suggest that healthy and active geriatric patients may benefit from THA to maximize postoperative function and quality of life,^32,33^ others have demonstrated no clinically significant benefit with an elevated risk of dislocations.^16,34^ Nevertheless, despite numerous studies comparing outcomes between HA and THA for geriatric femoral neck fractures, indications for either procedure remain unclear; a careful discussion of risks and benefits of both treatment modalities should be conducted with most patients, using shared decision making to guide treatment.

This study found a low 1-year (1.4%) and 5-year (2.3%) conversion rate of HA to THA. Previous studies have found similar HA conversion rates between 1% and 4%.^35-37^ The most common reason for conversion in our study was degeneration related to acetabular erosion (67.6% of conversions), which is cited by some studies as the most common reason for failed HA and conversion to THA,^19^ although aseptic loosening, periprosthetic femur fracture, and dislocation are also commonly cited modes of HA failure.^38,39^ The low conversion rate observed in our large study suggests that the commonly cited risks of acetabular erosion and periprosthetic fracture may actually be less concerning than what has been commonly suggested. It is important to note that we could not differentiate between cemented and noncemented HA, where cemented HA has a lower risk of periprosthetic fracture.^40^

Our multivariate analysis found that the youngest patients (age 65 to 74) were most at risk of conversion while increasing ECI increased odds of HA conversion. A retrospective analysis by Grosso et al. demonstrated that patients younger than 75 years had 4 times greater rate of conversion to THA when compared with patients aged 75 years or older (5.3% vs. 1.4%; *P* = 0.003).^19^ However, the HOPE trial, a prospective RCT comparing HA with THA for the management of femoral neck fractures among octogenarians and nonagenarians who were previously ambulatory with intact cognition, found that there were no differences between the two groups for postoperative complications, revisions, function, and quality of life at 2 years.^15^ Preoperative function and cognitive status can influence the decision between HA and THA, where HA is the preferred option for more frail, debilitated patients to minimize the dislocation rate.^18^

Although this is one of the largest national retrospective studies investigating risk factors of HA conversion to THA, there are several limitations. First, because the study was retrospective and based on administrative data, radiographic and functional outcomes could not be specifically assessed. Additional implant type (unipolar versus bipolar HA) and cemented versus noncemented stem could not be determined. In addition, the dataset did not capture mortality, and therefore, expired patients were excluded by virtue of requiring all patients to have 5 years of follow-up and thus capable of undergoing conversion during the study period; therefore, our cohort may be biased toward a healthier patient population. Hemiarthroplasty may be more commonly chosen for an older, more fragile patient population; thus, as a result, a notable portion of that patient cohort may have been excluded. Similarly, the patient demographics may bias the data in one direction, which may not match with the general patient population who suffered these injuries.

## Conclusions

Overall, there was a low conversion rate (2.3%) of HA to THA for those who were tracked for 5 years after HA for femoral neck fracture. Most of these were within the first year and related to degenerative causes. This low conversion rate is supportive that HA offers a durable answer for most patients for whom this treatment choice is pursued.
